# Pose-gait analysis for cetacean biologging tag data

**DOI:** 10.1371/journal.pone.0261800

**Published:** 2022-09-23

**Authors:** Ding Zhang, Kari Goodbar, Nicole West, Veronique Lesage, Susan E. Parks, David N. Wiley, Kira Barton, K. Alex Shorter

**Affiliations:** 1 Department of Mechanical Engineering, University of Michigan, Ann Arbor, MI, United States of America; 2 Dolphin Quest Oahu, Honolulu, HI, United States of America; 3 Fisheries and Oceans Canada, Ottawa, Canada; 4 Department of Biology, Syracuse University, Syracuse, NY, United States of America; 5 National Oceanic and Atmospheric Agency’s (NOAA) Stellwagen Bank National Marine Sanctuary, Scituate, MA, United States of America; University of Saint Andrews, UNITED KINGDOM

## Abstract

Biologging tags are a key enabling tool for investigating cetacean behavior and locomotion in their natural habitat. Identifying and then parameterizing gait from movement sensor data is critical for these investigations, but how best to characterize gait from tag data remains an open question. Further, the location and orientation of a tag on an animal in the field are variable and can change multiple times during a deployment. As a result, the relative orientation of the tag with respect to (wrt) the animal must be determined for analysis. Currently, custom scripts that involve species-specific heuristics tend to be used in the literature. These methods require a level of knowledge and experience that can affect the reliability and repeatability of the analysis. Swimming gait is composed of a sequence of body poses that have a specific spatial pattern, and tag-based measurements of this pattern can be utilized to determine the relative orientation of the tag. This work presents an automated data processing pipeline (and software) that takes advantage of these patterns to 1) Identify relative motion between the tag and animal; 2) Estimate the relative orientation of the tag wrt the animal using a data-driven approach; and 3) Calculate gait parameters that are stable and invariant to animal pose. Validation results from bottlenose dolphin tag data show that the average relative orientation error (tag wrt the body) after processing was within 11 degrees in roll, pitch, and yaw directions. The average precision and recall for detecting instances of relative motion in the dolphin data were 0.87 and 0.89, respectively. Tag data from humpback and beluga whales were then used to demonstrate how the gait analysis can be used to enhance tag-based investigations of movement and behavior. The MATLAB source code and data presented in the paper are publicly available (https://github.com/ding-z/cetacean-pose-gait-analysis.git), along with suggested best practices.

## Introduction

**Fig 1:**
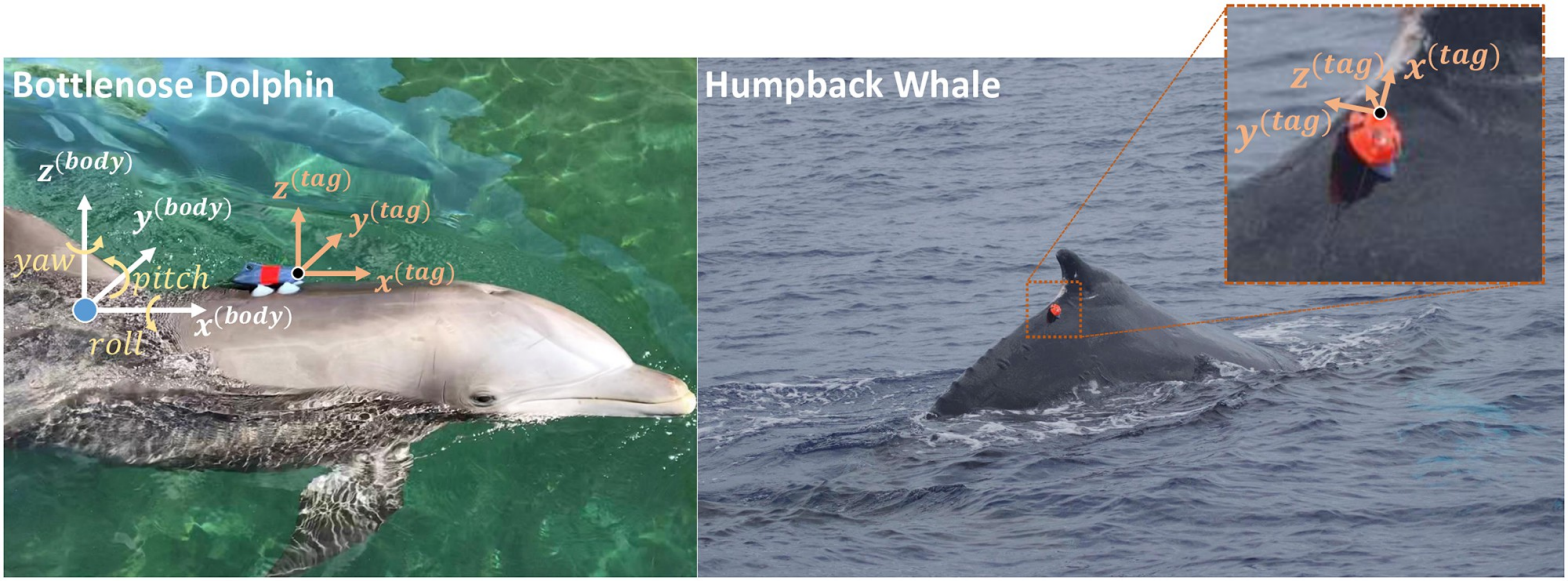
Biologging tags attached to a bottlenose dolphin (MTag, **left**) and a humpback whale (DTAG, **right**) along with the associated coordinate systems. Tag and body fixed coordinate systems may not be aligned when initially placed on an animal in the field (**right**). Further, tag orientation may shift during a deployment. Animal pose estimation requires knowledge about the relative orientation between the tag and animal. Note that a positive pitching angle corresponds to a negative rotation around the body fixed *y*-axis.

Biologging tags use sensors (e.g., accelerometers, magnetometers, gyroscopes, pressure, and hydrophones) to record data about animal movement, behavior, and the environment. Biologging tags are particularly important for the study of cetaceans because direct observation of these animals is often not possible. Tags are commonly used to study animal bioacoustics, biomechanics, and behavior [1–11]. Kinematic data from these tag systems have also been used to estimate animal locations to investigate the connection between environmental features and animal behaviors [12–22]. The key to many of these studies is the accurate estimation of animal pose (roll, pitch, and yaw, Fig 1).

Pose is essential for studies that require an estimated spatial trajectory of the animal or investigate locomotion and gait [5, 6, 9, 12–16, 23]. In the literature, pose is typically estimated using accelerometer and magnetometer data [1, 9]. When gyroscope data is available, filtering methods like [24] can be used to improve estimated animal pose using measurements of angular velocity to capture higher frequency motion. Swimming gait is essentially composed of a repetitive sequence of poses, but how to efficiently parameterize the gait of the animal from a pose sequence remains an open question. Studies in the literature commonly use pitch to identify and quantify gait via parameters like frequency and amplitude [5, 6, 9]. But these pitch-based gait descriptors are sensitive to the roll angle of the animal since pitch only measures the angle between the animal body’s caudal-rostral axis and the world’s horizontal plane. For example, the pitch of an animal derived from accelerometer data captured little information about the gait when the animal is fluking sideways (a 90-degree roll angle) because the body’s caudal-rostral axis remaining in the horizontal plane results in near-zero pitch measurements during locomotion. To better characterize gait, a measure wrt the animal’s body rather than the earth’s horizontal plane is needed. The measured angular rate from a gyroscope can be integrated to estimate the rotation angle wrt the animal itself. However, this estimation is subject to accumulated sensor error, and gyroscopes are unavailable in many tags. As such, it is essential to develop approaches that can address this in accelerometer-based estimates of orientation.

Because many cetacean tags use a suction cup-based attachment method, the location and orientation of the tag wrt the animal are variable [1, 25]. As a result, the relative orientation of the tag wrt the animal must be determined before the animal pose is calculated [1, 9, 26]. Further, even with best practices for deploying tags [25], the relative orientation between the tag and the animal can change during a deployment (e.g., the tag slides on the animal). We refer to this type of event as a tag **shift**. Identifying these shifts is essential for determining the relative orientation of the tag. Currently, relative orientation is determined manually or heuristically in a species-dependent manner using portions of data where the animal’s orientation can be inferred. For example, when an animal breathes at the surface (surfacing), it is assumed that the pose does not exhibit a significant roll. A surfacing event can be inferred from pressure data, and the roll estimate can then be corrected accordingly [1, 9, 26]. Tag orientation shifts can be identified by human inspection using sensor streams [1, 26], like accelerometer data, to identify features, such as an impact on the tag by another animal or object in the form of a surge in the signal. However, hydrodynamic forces may also affect the tag, resulting in a more gradual shift in the orientation. For these types of shifts, features in the sensor data can be harder to identify. Simulation and experimental studies have been used to estimate hydrodynamic forces that are acting on tags or imparted to the animals [27–29], but it is difficult to predict when the combined hydrodynamic and inertial forces resulting from animal motion will result in a shift. Currently, an automated approach to identify these relative changes in orientation is lacking.

To address these gaps, this paper presents an automated data processing pipeline (and software) to: (1) Identify time instances associated with occurrences of a relative orientation change between the tag and animal (i.e., tag shift); (2) Derive the relative orientation of the tag wrt the animal using a data-driven approach; and (3) Extract frequency and amplitude estimations of the gait that are stable and invariant to the animal’s pose by representing the animal’s high-frequency motion in its own low-frequency reference frame. The authors used biologging tag data from bottlenose dolphins, humpback whales, and beluga whales to validate and demonstrate the proposed methods. Specifically, data from bottlenose dolphins in a managed environment was augmented with simulated tag shifts to quantitatively evaluate the proposed method. Data from free-ranging humpback and beluga whales were used to demonstrate the method qualitatively. The proposed analysis approach will facilitate the use of biologging tags to study cetacean locomotion and behavior. Further, the proposed method can be used directly with cetacean data from any tag platform equipped with an accelerometer, magnetometer, and pressure sensor. Discussion and suggestions related to data processing best practices are also provided. The MATLAB source code and presented data are publicly available (https://github.com/ding-z/cetacean-pose-gait-analysis.git).

## Biologging tag platforms: MTag and DTAG

Methods presented in this paper are applicable to cetacean tag platforms equipped with an accelerometer, magnetometer, and pressure sensor. In this work, we assume that the sensors share (or can be converted to) a right-handed coordinate system. Biologging tag data collected with MTags and DTAGs were used in this work (Fig 1). Specifically, MTag data from bottlenose dolphins and DTAG data from humpback and beluga whales are used to demonstrate the proposed approach. Both tag systems use suction cups to secure tag electronics to the animal. MTag sensors include a 9 DOF (Degrees of Freedom) IMU (Inertial Measurement Unit) with an accelerometer, gyroscope, magnetometer, and additional sensors to record temperature, pressure, and speed [30]. The tags recorded IMU data at 50 Hz and all other sensors at 10 Hz. The DTAG platform [1] (Fig 1-Right) contains accelerometers, magnetometers, a pressure sensor, and hydrophones, but no gyroscope. DTAG accelerometer and magnetometer data were collected at 250 Hz.

## Initial processing and assumptions

**Fig 2:**
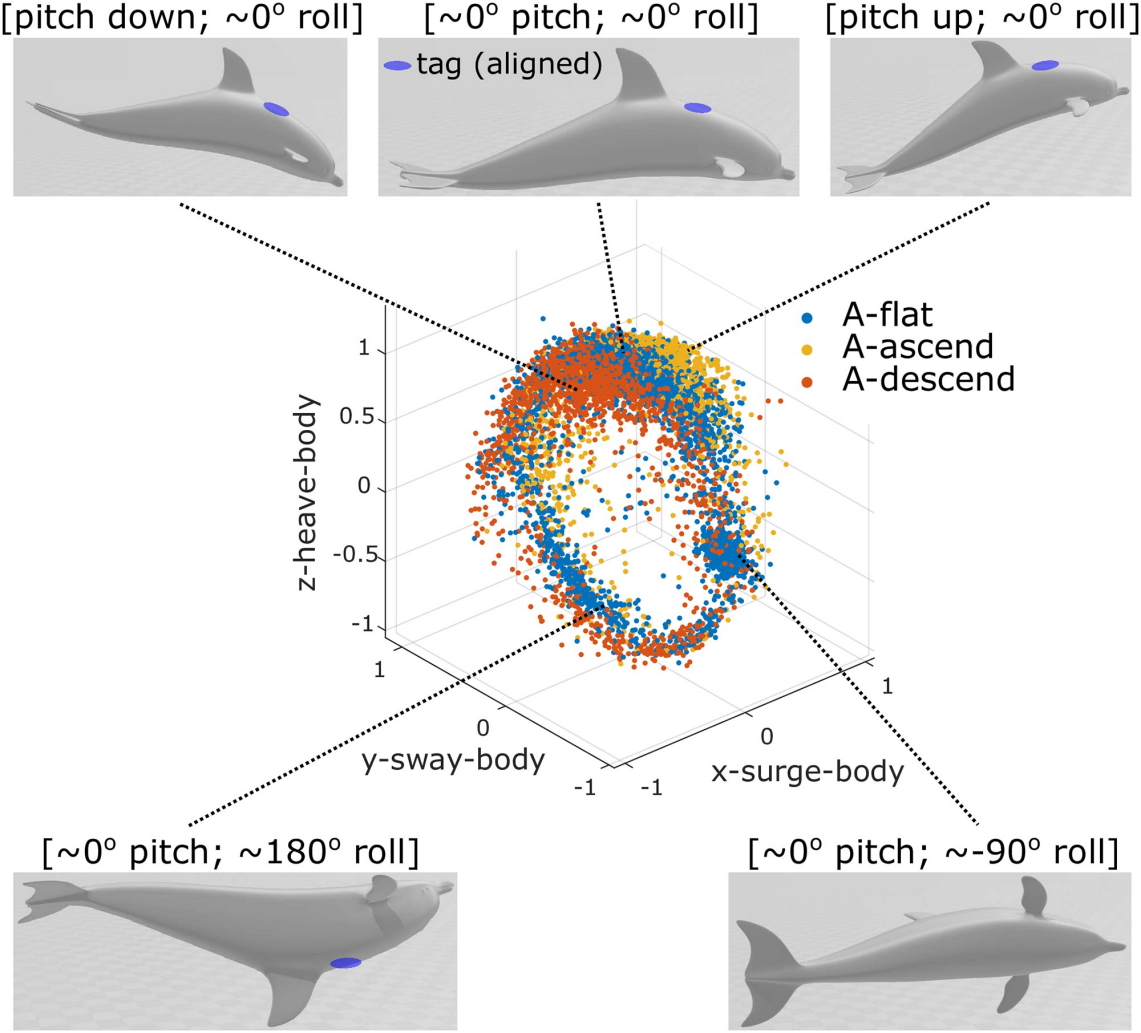
An orientation sphere for a bottlenose dolphin with a biologging tag aligned with the animal’s body. Each data point represents an acceleration measurement at one time instance during swimming (i.e., one tag data sample) and is clustered into groups labeled ‘flat,’ ‘ascend,’ or ‘descend’ based on measured vertical speed from pressure data. As the animal changes pose, the location of the gravitational acceleration on the sphere also changes. The swimming motion of an animal is composed of a sequence of poses that correspond to areas on the orientation sphere. The top 3 poses illustrate a shallow diving cycle with a neutral roll.

The proposed work estimates animal pose using biologging data (from a 3-axis accelerometer, 3-axis magnetometer, and pressure/depth sensor) and features in the kinematic data created during cetacean swimming. Tag accelerometers measure the acceleration due to both gravity and animal motion. As the animal changes pose, the gravitational force measured by each component axis of the accelerometer changes in the tag coordinate frame, while the magnitude of the total signal stays constant. To obtain the gravitational component of the signal in the tag coordinate frame (***A***^(*tag*)^), we use a moving average window of 0.5 seconds to low-pass filter the accelerometer data. The magnitude and direction of gravitational acceleration in the world frame are known and will be used with the tag measurements to help estimate orientation. Next, ***A***^(*tag*)^ is used together with the vertical speed of the animal calculated from the pressure measurements to cluster data and visualize animal pose during swimming gait. We refer to these plots as **orientation spheres** (Fig 2). These plots capture body pose in contrast with the *o-sphere* presented in [31] that was used to visualize animal head movement.

The proposed automated shift detection and orientation correction approach requires three key assumptions about the pose of the animals during swimming gait. **First**, and most important, is that the mode of the roll angle distribution is neutral (0 degree angle) for cetacean swimming. **Second**, the animals maintain a positive pitch when ascending and a negative pitch when descending. **Third**, the same movement pattern is assigned before and after a shift event. For example, if the tag shifts while the animal is fluking during an ascent, we assume the same movement pattern before and after the tag shift. These assumptions are based on subject matter expertise and direct observations from experiments. The validity of the assumptions, the sensitivity of the method, and the identification of cases where these assumptions do not hold will be discussed.

## Nonparametric tag shift detection

**Fig 3:**
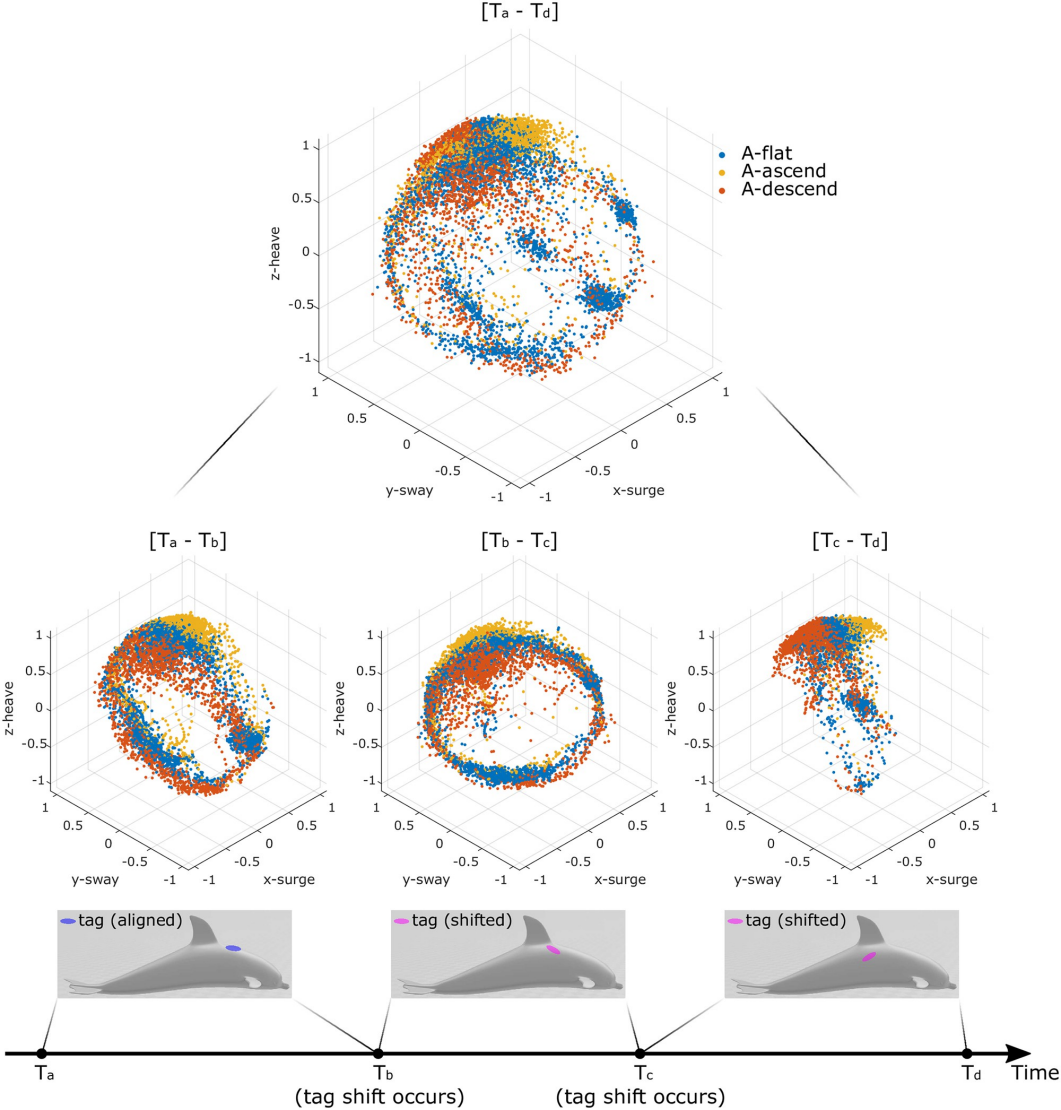
Orientation spheres for a bottlenose dolphin during three sections of a deployment. In this example, the tag shifted twice, *T*_*b*_ and *T*_*c*_, respectively. The proposed approach will 1) detect shift instances *T*_*b*_ and *T*_*c*_, and 2) generate the orientation transformation for each segment to align the tag and body reference frames (i.e., [*T*_*a*_-*T*_*b*_], [*T*_*b*_-*T*_*c*_], and [*T*_*c*_-*T*_*d*_]).

Forces acting on the tag can result in changes to the relative orientation between the tag and animal, creating multiple temporal segments with different relative orientations during the same deployment, Fig 3. In our approach, tag shifts are detected by identifying time instances when the patterns in the sensor data created by animal movement change significantly. *Abnormal* segments in the dataset are located by comparing distributions from different data segments from the same deployment. Comparisons are performed on individual data points without prior/expert knowledge of the data distribution. While discrete changes in the movement patterns measured by the tag are most often the result of hydrodynamic forces or contact with other animals, changes in swimming gait can also create detectable chances in the data streams.

We will first describe our approach to a constrained subproblem: identification of a tag shift within a given section of data *S* that contains at most one shift. For this subproblem, we divide *S* equally into temporally adjacent segments *S*_1_ and *S*_2_, each with duration *D*_*s*_ = 10 minutes. We assume that a shift occurs in either *S*_1_ or *S*_2_, but not both. Without loss of generality, we will assume the shift lies in *S*_2_. When a shift lies in *S*_2_, the data in *S*_1_ are used to form a comparison template for identifying the time instance *t*_2_ when the shift occurs. Once *t*_2_ is identified, *S*_2_ can be further divided into subsegments $S_{2}^{\left(t<t_{2}\right)}$ (i.e., before the shift) and $S_{2}^{\left(t\geq t_{2}\right)}$ (i.e., after the shift). We know that $S_{2}^{\left(t<t_{2}\right)}$ will share the same distribution as *S*_1_ and $S_{2}^{\left(t\geq t_{2}\right)}$ will diverge from *S*_1_.

**Fig 4:**
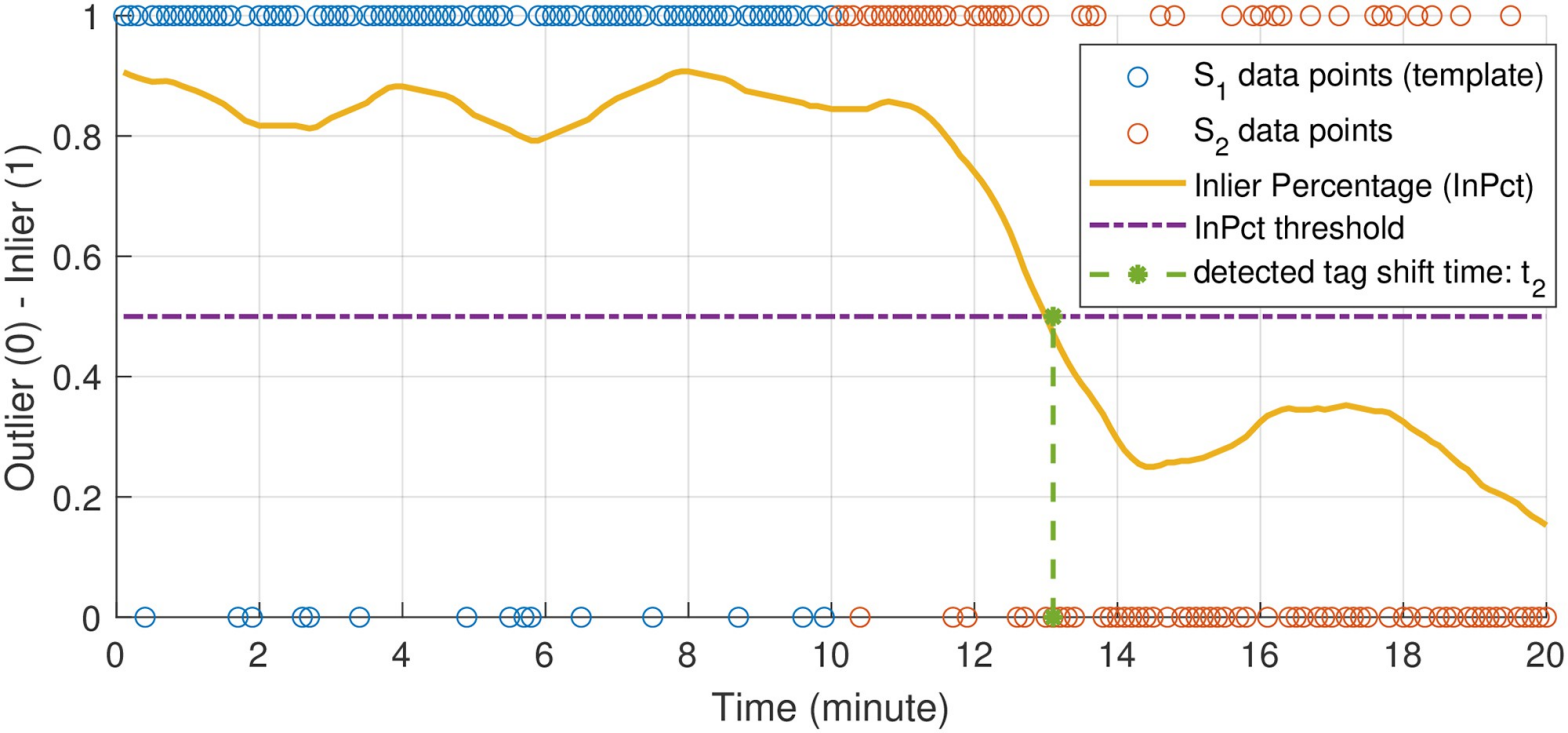
Conceptual illustration of determining tag shift time *t*_2_ in data segment *S*_2_ using data segment *S*_1_ as a template. Segment duration time is *D*_*s*_ = 10 minutes. Data points in *S*_2_ are checked against the template distribution (*S*_1_) to decide whether they are an inlier (1) or an outlier (0) of the template. Inlier percentage (*InPct*) is then calculated over time, and the tag shift time is determined by finding when *InPct* drops below an empirically defined threshold. If *t*_2_ does not exist or it is too close to the boundary between *S*_1_ and *S*_2_, this process is repeated to determine if there is a shift in *S*_1_.

To specifically identify the time stamp of *t*_2_ in *S*_2_, we use the orientation sphere data in *S*_1_ as a template. For each data point in *S*_2_ (e.g., any point in Fig 2), we find its *K* nearest Euclidean spatial neighbors in *S*_1_ and compute the average distance between this point and its Euclidean spatial neighbors. In this work, *K* = 30 was determined based on a qualitative assessment of the distribution of points. If the resulting distance is within a defined threshold (e.g., 0.1 g based on a qualitative assessment of the distribution of points), the data point in *S*_2_ is considered an **inlier** of *S*_1_; otherwise, it is classified as an **outlier**. After all of the points in *S*_2_ are assigned with a value of 1 (inlier) or 0 (outlier), a temporal moving average filter is applied to obtain a local inlier percentage (*InPct*) value. As illustrated in Fig 4, the time instance *t*_2_ is found by identifying the first time *InPct* drops below a pre-defined threshold. If *InPct* never drops below this threshold, or *t*_2_ is too close to the boundary between *S*_1_ and *S*_2_, then the shift could be contained within *S*_1_ instead of *S*_2_. The same procedure can then be applied to identify a shift *t*_1_ in *S*_1_.

**Fig 5:**
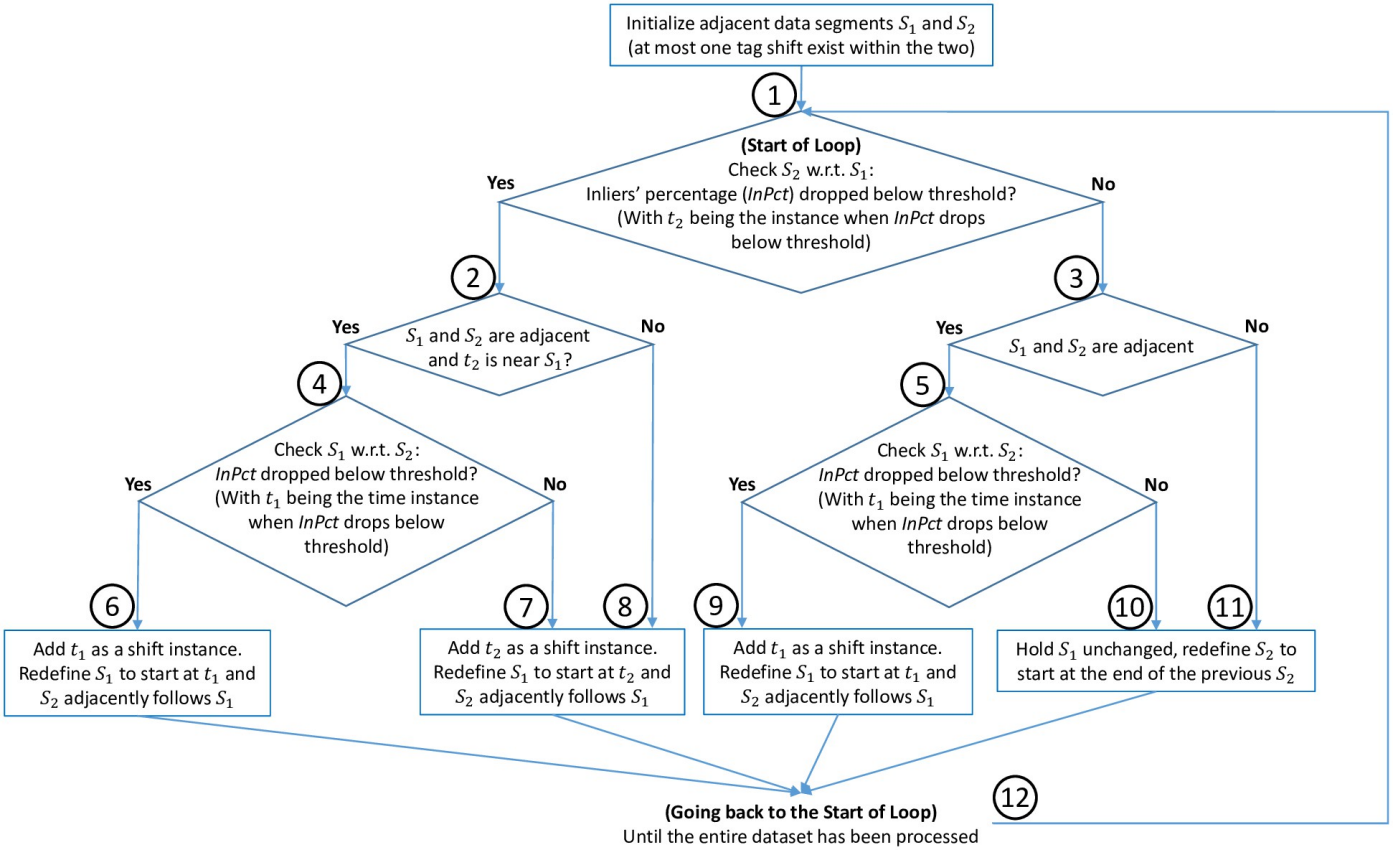
Tag shift detection algorithm. Each branch is marked by a circled number (1 to 12) to aid discussion in the text.

With the strategy defined above to find a shifting instance within two segments, *S*_1_ and *S*_2_, an algorithm for finding all tag shifts is presented in Fig 5. Based on the procedure described above, the algorithm starts with adjacent data segments *S*_1_ and *S*_2_. The segments share a fixed empirically defined duration *D*_*s*_ that reflects the expected minimum tag shift interval (e.g., *D*_*s*_ = 10 minutes). If neither segment contains a shift, then *S*_1_ remains unchanged, and the procedure continues by checking the next data segment, relabeled as *S*_2_, and so on. Once a shift is detected, both segments will be redefined such that *S*_1_ starts from the identified shift and *S*_2_ adjacently follows (branches 6 to 9 in Fig 5).

If *S*_2_ contains a shift instance *t*_2_ (branch 2), and *t*_2_ does not lie within 3 minutes of the *S*_1_ → *S*_2_ segment transition, then *t*_2_ is recorded as a shift. However, if *t*_2_ lies near the *S*_1_ (branch 4), the algorithm will check segment *S*_1_ to determine whether the shift initiates in *S*_1_. Depending on the search results, either *t*_1_ or *t*_2_ will be recorded as the shift (branches 6 & 7). If *S*_2_ is redefined (i.e., *S*_1_ and *S*_2_ are not adjacent), the algorithm only searches for a shift in *S*_2_ (branches 8 & 11). These steps are repeated until the entire dataset has been searched (branch 12).

## Pattern-based orientation correction

For a given segment of data that does not contain a tag shift but has an unknown tag-animal configuration, the tag data from the accelerometer and magnetometer (and gyroscope, if equipped) can be rotated to transform the data from tag coordinates to the animal’s body coordinates. In this work, such a rotation is found by matching the measured general motion pattern, i.e., orientation sphere, to an assumed one. Depth measurements are used to group the gravity measurements in the tag frame, ***A***^(*tag*)^, into three clusters: $A_{flat}^{\left(tag\right)}$, $A_{ascend}^{\left(tag\right)}$ and $A_{descend}^{\left(tag\right)}$, in accordance with the animal swimming horizontally, ascending, and descending, respectively.

**Fig 6:**
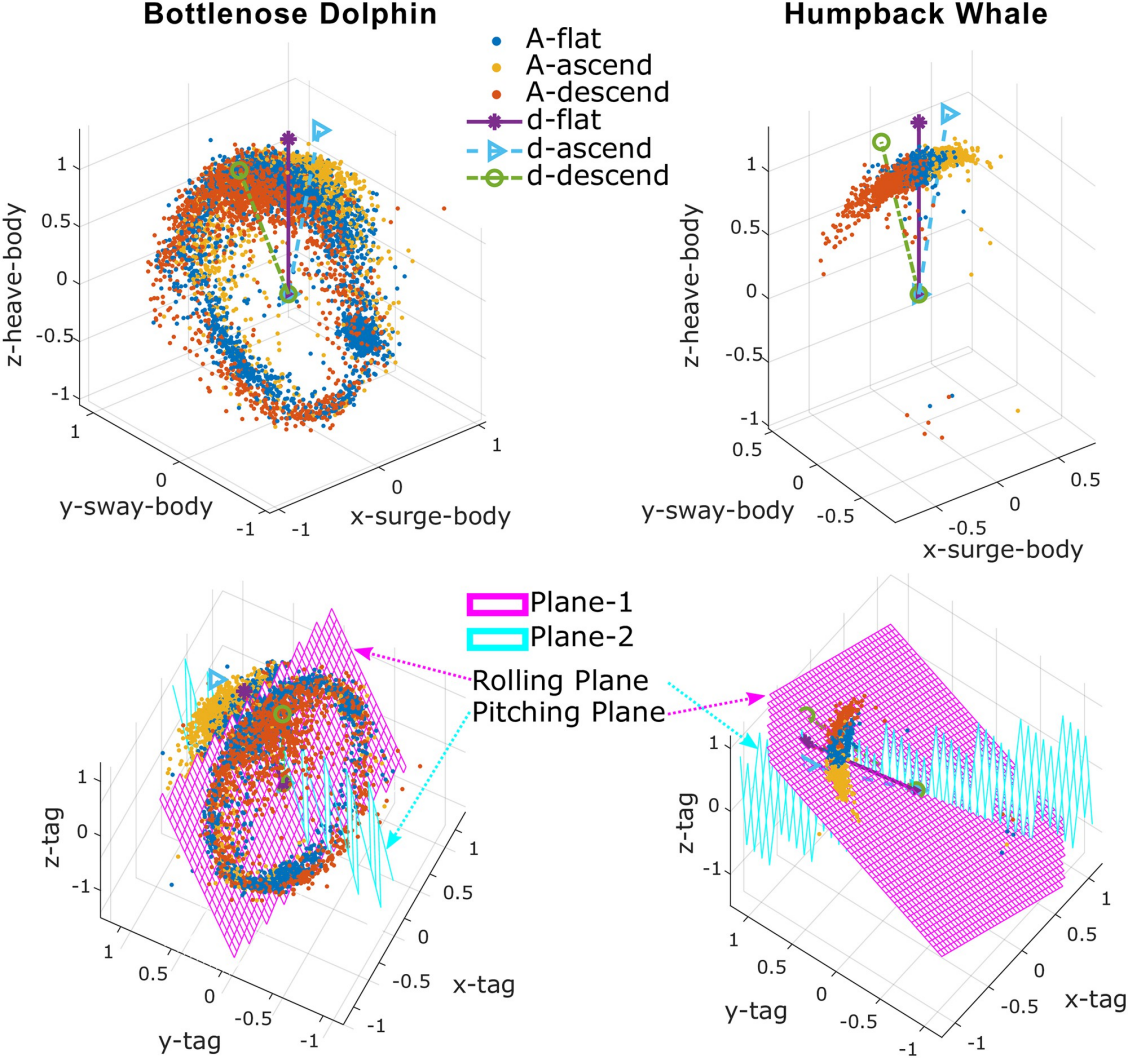
**Orientation spheres** for a data segment of a bottlenose dolphin (**left column**) and a humpback whale (**right column**). The plots provide a visualization of the orientation correction method applied to an uncorrected data segment to find the dominant directions in the tag’s coordinates (**bottom row**). The prevailing directions can then be mapped to their assumed directions in the body coordinate frame (**top row**).

We further define the **dominant direction** as the gravity measurement direction associated with the most common (mode) pose of the animal for each of the three swimming conditions: $d_{flat}^{\left(tag\right)}$, $d_{ascend}^{\left(tag\right)}$, and $d_{descend}^{\left(tag\right)}$. With the assumptions about the general motion of these animals provided in Section: Initial Processing and Assumptions, the most common pose of a cetacean under each of the three swimming conditions is assumed to be known. In particular, $d_{flat}^{\left(tag\right)}$ corresponds to the gravity measurement when the animal has zero pitch and zero roll, while $d_{ascend}^{\left(tag\right)}$ corresponds to positive pitch and zero roll, and $d_{descend}^{\left(tag\right)}$ corresponds to negative pitch and zero roll. Specifically, $d_{flat}^{\left(body\right)}$ is aligned with the body’s *z*-axis (*z*^(*body*)^), and $d_{ascend}^{\left(body\right)}$ lies in the first quadrant of the plane formed by the *x*^(*body*)^ and *z*^(*body*)^ axes, while $d_{descend}^{\left(body\right)}$ lies in the second quadrant (Fig 6-Top Row). With these labels, ***d***_*flat*_ is used to identify the *z*^(*body*)^ direction of the animal, while ***d***_*ascend*_ and ***d***_*descend*_ are used to identify the forward direction (*x*^(*body*)^) of the animal; magnitude and relative angle of these three vectors are not considered in the orientation correction process. If $d_{flat}^{\left(tag\right)}$, $d_{ascend}^{\left(tag\right)}$ and $d_{descend}^{\left(tag\right)}$ can be found in the tag coordinates (Fig 6-Bottom Row), the rotations that map them to $d_{flat}^{\left(body\right)}$, $d_{ascend}^{\left(body\right)}$ and $d_{descend}^{\left(body\right)}$ can be determined and applied to map additional data from the tag to animal body coordinate frame (Fig 6-Top Row).

To determine the dominant directions within uncorrected data, two perpendicular planes are fitted to ***A***^(*tag*)^ using the RANSAC algorithm [32] (Fig 6-Bottom Row), which is particularly robust to outliers and imbalanced data as compared to least square-based approaches. The two planes correspond to two physical motions: rolling with zero pitch for the **rolling plane** and pitching with zero roll for the **pitching plane**.

Firstly, plane-1 (through the origin) is fitted to the most significant distribution in the data, which could be either $A_{flat}^{\left(tag\right)}$ (e.g., Fig 6-Bottom Left, plane-1 is a **rolling plane**) or $A_{ascend}^{\left(tag\right)}$ and $A_{descend}^{\left(tag\right)}$ (e.g., Fig 6-Bottom Right, plane-1 is a **pitching plane**), depending on the animal and environment. Secondly, plane-2 (through the origin) is fitted to the rest of the data under the constraint that it is perpendicular to plane-1. The toolbox can assign the roles of **rolling plane** and **pitching plane** to the two planes automatically.

The intersection line of the two planes is colinear with $d_{flat}^{\left(tag\right)}$ while both $d_{ascend}^{\left(tag\right)}$ and $d_{descend}^{\left(tag\right)}$ lie in the **pitching plane**. If we further define $a_{flat}^{\left(tag\right)}$, $a_{ascend}^{\left(tag\right)}$, and $a_{descend}^{\left(tag\right)}$ to be the average values of $A_{flat}^{\left(tag\right)}$, $A_{ascend}^{\left(tag\right)}$, and $A_{descend}^{\left(tag\right)}$, then projecting $a_{flat}^{\left(tag\right)}$ onto the intersection line produces $d_{flat}^{\left(tag\right)}$, while projecting $a_{ascend}^{\left(tag\right)}$ and $a_{descend}^{\left(tag\right)}$ onto the **pitching plane** returns $d_{ascend}^{\left(tag\right)}$ and $d_{descend}^{\left(tag\right)}$ (Fig 6-Bottom Row), respectively.

With $d_{flat}^{\left(tag\right)}$, $d_{ascend}^{\left(tag\right)}$, and $d_{descend}^{\left(tag\right)}$ identified in the tag coordinates, the data can be put into the animal’s body coordinates (i.e., ***A***^(*tag*)^ → ***A***^(*body*)^) with two rotations: first, a rotation (***R***_1_) to align $d_{flat}^{\left(tag\right)}$ with the body’s *z*-axis (*z*^(*body*)^) and second, a rotation (***R***_2_) around the *z*-axis so ***d***_*ascend*_ and ***d***_*descend*_ are on the *x*-*z* plane with ***d***_*ascend*_ on the positive *x*-axis side and ***d***_*descend*_ on the negative *x*-axis side (Fig 6-Top Row). Other data are mapped from tag coordinates to body coordinates using ***R***_1_ and ***R***_2_:

$$\begin{array}{cc} A^{\left(body\right)} & =A^{\left(tag\right)}R_{1}R_{2}, \end{array}$$ (1)

$$\begin{array}{cc} M^{\left(body\right)} & =M^{\left(tag\right)}R_{1}R_{2}, \end{array}$$ (2)

with ***A***^(*body*)^ and ***M***^(*body*)^ being the transformed (animal’s body coordinates) data from the accelerometer and magnetometer.

## Animal pose calculation and gait characterization

Roll, pitch, and yaw are widely used to describe the pose of animals [1, 9]. In this work we are computing them in a conventional [1] way using the transformed (animal’s body coordinates) data from accelerometer ***A***^(*body*)^ and magnetometer ***M***^(*body*)^. Define ***R***_*x*_(*α*) to be the rotation matrix for rotating the data about the *x*-axis with angle *α*. And similarly ***R***_*y*_(*β*) about the *y*-axis with angle *β* and ***R***_*z*_(*γ*) about the *z*-axis with angle *γ*. For time instance *t*, the 3-axes body coordinate accelerometer and magnetometer data are represented as $A_{t}^{\left(body\right)}=\left[A_{xt}^{\left(b\right)},A_{yt}^{\left(b\right)},A_{zt}^{\left(b\right)}\right]$ and $M_{t}^{\left(body\right)}=\left[M_{xt}^{\left(b\right)},M_{yt}^{\left(b\right)},M_{zt}^{\left(b\right)}\right]$. Roll, pitch, and yaw are calculated as:

$$\begin{array}{c} roll_{t}=\text{arctan}(A_{zt}^{\left(b\right)}/A_{yt}^{\left(b\right)}), \end{array}$$ (3)

$$\begin{array}{c} pitch_{t}=\text{arcsin}(A_{xt}^{\left(b\right)}/norm\left(\left[A_{xt}^{\left(b\right)},A_{yt}^{\left(b\right)},A_{zt}^{\left(b\right)}\right]\right), \end{array}$$ (4)

$$\begin{array}{c} \left[M_{xt}^{\left(2\right)},M_{yt}^{\left(2\right)},M_{zt}^{\left(2\right)}\right]=\left[M_{xt}^{\left(b\right)},M_{yt}^{\left(b\right)},M_{zt}^{\left(b\right)}\right]R_{x}\left(roll_{t}\right)R_{y}\left(-pitch_{t}\right), \end{array}$$ (5)

$$\begin{array}{c} yaw_{t}=-\text{arctan}(M_{yt}^{\left(2\right)}/M_{xt}^{\left(2\right)}), \end{array}$$ (6)

where we note that the body coordinate axes, *x*^(*body*)^, *y*^(*body*)^, and *z*^(*body*)^, are defined to be pointing forward, leftward, and upward wrt the animal’s body, respectively (Fig 1).

Let a set of moving coordinates {*x*^(*move*)^, *y*^(*move*)^, *z*^(*move*)^} be initially aligned with the earth’s inertial coordinates {*x*^(*inertial*)^, *y*^(*inertial*)^, *z*^(*inertial*)^}. For the inertial coordinates, we define *x*^(*inertial*)^ pointing to magnetic north, *z*^(*inertial*)^ pointing vertically upwards, and *y*^(*inertial*)^ following the right hand rule to point to magnetic west. Yaw, pitch, and roll rotate the moving coordinates from the inertial coordinates to the animal’s body coordinates in 3 steps:

1. Yaw corresponds to a positive rotation around the moving coordinates initial *z*^(*move*)^ axis where *x*^(*move*)^ is aligned with the projection of the animal’s *x*^(*body*)^ axis on the earth’s horizontal plane.
2. Pitch measures the angle between the body coordinates *x*^(*body*)^ axis and the horizontal earth plane, rotates around the moving coordinate *y*^(*move*)^ axis, and ensures that *x*^(*move*)^ is aligned with the animal’s *x*^(*body*)^ axis. Note that a positive pitch (i.e., animal head up) corresponds to a negative rotation around *y*^(*move*)^, with *y*^(*body*)^ defined pointing to the left of the animal.
3. Roll represents a positive rotation around the moving coordinate *x*^(*move*)^ axis (which is now aligned with the animal’s *x*^(*body*)^ axis). This brings the moving coordinate *y*^(*move*)^ axis from a horizontal to the animal’s *y*^(*body*)^ axis. After this step, *x*^(*move*)^, *y*^(*move*)^, and *z*^(*move*)^ should be aligned with *x*^(*body*)^, *y*^(*body*)^, and *z*^(*body*)^, respectively.

An estimated pitch is commonly used to describe the fluking gait of the animal [5, 6, 9]. However, because pitch only measures the angle between the animal’s *x*^(*body*)^ axis and the horizontal earth plane, it does not capture gait characteristics well when the animal is rolling. This is particularly egregious when the animal is fluking with a roll angle of ±90°, where the fluking motion does not show up in the pitch measurement. Similarly, the characterization of fluking using a high-frequency component present in acceleration measurements can be affected by the orientation of the animal [33]. The amplitude of the oscillations measured by the accelerometer decreases during periods of fluking when the animal is rolled on its side. The pitching angle wrt the animal’s *y*^(*body*)^ axis can be estimated by integrating angular velocity measurements from gyroscopes. While these angular estimates are subject to sensor drift, the gait parameters calculated using this approach are not affected by the orientation of the animal. Despite this potential advantage, accelerometer-based approaches are more common because most tag platforms do not include gyroscopes. In this work, our approach calculates frequency and amplitude estimates of gait that are stable and invariant to animal pose. This is achieved by representing the animal’s high-frequency motion in its own low-frequency reference frame, with details presented below.

In the original axes, {*x*^(*body*)^, *y*^(*body*)^, *z*^(*body*)^} move wrt the earth’s inertial coordinate frame following the animal’s motion (e.g., **high** frequency fluking and **low** frequency transitions from ascending to descending). We now define a new set of coordinates $\left\{{\bar{x}}^{\left(body\right)},{\bar{y}}^{\left(body\right)},{\bar{z}}^{\left(body\right)}\right\}$ that follow the **low** frequency motion of the animal, which represents the neutral pose of the animal during high frequency periodic motion. Then, $\left\{{\bar{x}}^{\left(body\right)},{\bar{y}}^{\left(body\right)},{\bar{z}}^{\left(body\right)}\right\}$ can be used to characterize the animal’s **high** frequency motion wrt the animal itself, rather than the earth’s inertial coordinates.

Specifically, representing *x*^(*body*)^ in $\left\{{\bar{x}}^{\left(body\right)},{\bar{y}}^{\left(body\right)},{\bar{z}}^{\left(body\right)}\right\}$ returns the dynamic heading of the animal wrt its own neutral body pose. For example, an animal’s head tilting up and down wrt its neutral body pose would result in the vector *x*^(*body*)^ swinging in the ${\bar{x}}^{\left(body\right)}$-${\bar{z}}^{\left(body\right)}$ plane. We now refer to the vector *x*^(*body*)^, represented in $\left\{{\bar{x}}^{\left(body\right)},{\bar{y}}^{\left(body\right)},{\bar{z}}^{\left(body\right)}\right\}$, as the **vector of dynamic pose** (***V***_*dp*_):

$$\begin{array}{c} V_{dp(t)}=\underset{3D\;heading}{\underset{︸}{\left[1,0,0\right]R_{y}\left(-pitch_{t}\right)R_{z}\left(yaw_{t}\right)}}\underset{low\;frequency\;body\;map}{\underset{︸}{R_{z}^{-1}\left({\bar{yaw}}_{t}\right)R_{y}^{-1}\left(-{\bar{pitch}}_{t}\right)R_{x}^{-1}\left({\bar{roll}}_{t}\right)}}, \end{array}$$ (7)

where [1, 0, 0] is an unit vector pointing forward while ${\bar{roll}}_{t}$, ${\bar{pitch}}_{t}$ and ${\bar{yaw}}_{t}$ are the low-pass filtered roll, pitch and yaw values at time *t*. We note that a positive animal pitch is a negative rotation around the *y*-axis under the current axes definition. In (7), the first section determines the animal’s current 3D heading (*x*^(*body*)^) in the inertial coordinates and the second section maps *x*^(*body*)^ from the inertial coordinates to the low-frequency body coordinates $\left\{{\bar{x}}^{\left(body\right)},{\bar{y}}^{\left(body\right)},{\bar{z}}^{\left(body\right)}\right\}$.

The resulting ***V***_*dp*_ gives the dynamic 3D heading of the animal wrt the animal’s low-frequency body pose. With this transition, the fluking motion of cetaceans will be observed in the ${\bar{x}}^{\left(body\right)}$-${\bar{z}}^{\left(body\right)}$ plane (i.e., **pitching plane**). We further define **dynamic pitch** (*pitch*_*dp*_) and **dynamic yaw** (*yaw*_*dp*_) to be the angles between ***V***_*dp*_ and the *x*-*y* and *x*-*z* planes, respectively:

$$\begin{array}{c} pitch_{dp(t)}=\text{arcsin}(V_{dpz(t)}/norm\left(V_{dp(t)}\right)), \end{array}$$ (8)

$$\begin{array}{c} yaw_{dp(t)}=\text{arcsin}(V_{dpy(t)}/norm\left(V_{dp(t)}\right)), \end{array}$$ (9)

with ***V***_*dp*(*t*)_ = [*V*_*dpx*(*t*)_, *V*_*dpy*(*t*)_, *V*_*dpz*(*t*)_] at time *t*. Dynamic pitch and dynamic yaw approximate the animal’s dynamic angle changes around its ${\bar{y}}^{\left(body\right)}$ and ${\bar{z}}^{\left(body\right)}$ axes, respectively. In this work, *pitch*_*dp*_ is of particular importance as it provides a measure of gait for a cetacean.

An animal’s per-stroke fluking period and amplitude are calculated by automatically locating and processing the positive and negative peaks in *pitch*_*dp*_ (findpeaks function in Matlab and parse_gait function in the proposed toolbox with default settings included), which enables a wide variety of further analyses (e.g., investigating the animal’s fluking frequency and amplitude during fast diving in contrast to slow diving). Meanwhile, data segments that do not contain any peaks are marked as passive gait behaviors (e.g., gliding).

## Validation

Validation objectives in this work included: (**1**) With what precision can the proposed method identify tag shifts? (**2**) Can the proposed method identify a data correction map to compensate for misalignments between the tag and animal? (**3**) How accurate is the calculated animal pose compared to the ground truth? (**4**) Is the proposed method sensitive enough to detect a small shift in the tag? (**5**) What is the impact of the defined segment duration *D*_*s*_ on the shift detection performance? (**6**) Can the proposed technique be applied to different cetaceans?

Biologging tag (MTag) data collected from bottlenose dolphins (*Tursiops truncatus*) under human care in Dolphin Quest Oahu, Hawaii, were used to validate the proposed approach. The biologging tag was aligned with the animal and attached 20 cm behind the blowhole non-invasively via four silicone suction cups (Fig 1-Left). A total of 18 datasets, with an average duration of 87 (±23) minutes, were included for the validation. These datasets were selected for analysis because the orientation of the tags remained constant during the ∼26 hours of data collection.

As a correctly aligned tag measurement differs from a misaligned tag primarily by a rotation to the data (i.e., a change of coordinates), we injected artificial rotations to the initially aligned data to simulate the effects of tag shifts. For validation purposes, randomly designed rotations were applied to randomly defined data segments to simulate the effects of a tag shift. Approximately 100 random simulations were conducted for each of the 18 datasets. The dataset was randomly broken into *k*+ 1 segments for each simulation run, with the random integer *k* ranging between 1 and 6, representing the number of injected tag shifts. A random rotation, with both random direction and magnitude (in degrees), was then applied to each data segment. The method used an empirically specified segment duration setting *D*_*s*_ = 10 minutes for shift detection. As a point of comparison and verification, shift detection was also performed over the original datasets to investigate the detector’s performance when no shifts existed in the datasets.

For tag shift detection (**Objective** **1**), we define the **precision error** of the detection as the absolute time difference between the detected shift instance and the nearest injected shift instance. Further, the detection is identified as a **positive detection** if the precision error is within 300 seconds (5 minutes). Otherwise, the detection is considered as a **negative detection**. **Precision** and **recall** values are calculated as:

$$\begin{array}{c} \text{precision}=\frac{\text{positive}\;\text{detections}}{\text{all}\;\text{detections}}, \end{array}$$ (10)

$$\begin{array}{c} \text{recall}=\frac{\text{positive}\;\text{detections}}{\text{injected}\;\text{shifts}}. \end{array}$$ (11)

To evaluate whether the rotations have been corrected using a correction map (**Objective** **2**), poses (roll, pitch, and yaw) calculated from **corrected** simulated data segments (condition ***A***) were compared with poses calculated from the original data segments (no injected rotation shifts—condition **B**).

As a further means of evaluating the corrections, we also investigated the performance of the baseline pose calculation method described in this manuscript (**Objective** **3**), poses calculated using accelerometer and magnetometer data—condition **B**, compared to poses calculated using a gradient descent based filtering approach [24] (referred to as *Madgwick*’s approach), which used accelerometer, magnetometer, and gyroscope data (condition **C**). Results from the more established *Madgwick*’s approach are considered to provide a ground truth comparison.

To assess the sensitivity of the proposed shift detection method (**Objective** **4**), we performed repeated computational experiments by injecting simulated tag shifts, with the simulated tag rotations exhibiting random directions with **fixed** degrees for each run. Specifically, 50 runs were applied to each dataset for a given fixed degree. For each run, *k*+ 1 rotations were injected into the dataset, with each rotation given a random direction. Average precision and recall in detecting the shifting instances were calculated for each fixed degree over all datasets and runs. Additionally, the absolute angle differences between poses calculated from corrected simulated data segments (condition ***A***) and poses calculated from the original data segments (condition **B**) were calculated for each fixed angle.

All computation experiments were repeated to assess the impact of segment duration *D*_*s*_ on the shift detection performance (**Objective 5**). Different *D*_*s*_ choices were evaluated against a varying number of injected tag shifts (i.e., *k*) to explore the relationship between user-specified segment duration *D*_*s*_ and the expected tag shift interval (determined by *k*). For each {*D*_*s*_, *k*} combination, 50 random runs were conducted on the datasets to calculate the average precision and recall of shift detection.

In addition to the bottlenose dolphin datasets, DTAG [1] data from a humpback whale (22.03 hours) and a beluga whale (2.25 hours) collected in the wild were included to evaluate the effectiveness of the shift detection and correction methods applied to different cetaceans and demonstrate gait analysis capability across datasets (**Objective 6**). Evaluation of the method’s performance on the datasets from free-ranging animals, where ground truth was unavailable, was examined qualitatively by inspecting the depth, roll, pitch, and orientation sphere plots after data correction.

The study protocols were approved by the University of Michigan Animal Welfare Committee (IACUC, #PRO00008825), the US National Marine Fisheries Service (NMFS, #18059), and the Canadian Council on Animal Care (#17-4, 18-3, 18-3B).

## Results

Across all 100 runs over the 18 datasets (**Objective** **1**), the average precision for shift instance detection was 0.87, the average recall was 0.89, and the average precision error was 37.5 (±18.4) seconds. A total of three false positives (i.e., detections that did not correspond to a tag shift) were generated by the shift detection algorithm, corresponding to a false detection rate of one occurrence per 8.7 hours. Poses (roll, pitch, and yaw) calculated from **corrected** data segments (condition ***A***) were compared with poses calculated from the original data segments (condition **B**, **Objective** **2**), with results shown in Table 1-Left. The average pose angle errors and standard deviations were within 11 degrees for all poses. Meanwhile, poses calculated in condition **B** were further compared with poses calculated using *Madgwick*’s approach [24] (condition **C**, **Objective** **3**, Table 1-Right). The average errors were within 3 degrees, and the standard deviations were within 6 degrees.

**Table 1:** Pose angle differences across different conditions. Condition ***A*** represents the pose calculated from simulated data after the simulated tag shifts have been corrected using the proposed method. Condition **B** presents the pose calculated from the original data (without tag shift). Condition **C** represents pose calculated from the original data (without tag shift) using Madgwick’s filter [24], which involves the additional use of a gyroscope. Direct difference (e.g., *A* − *B*) is used to detect a bias in the difference, while the absolute difference (e.g., |*A* − *B*|) returns the magnitude of the difference. Pose angle differences are in degree. Each cell gives a mean ± standard deviation.

Pose	A vs. B		B vs. C	
	*A* − *B*	|*A* − *B*|	*B* − *C*	|*B* − *C*|
***Roll***	2.4 ± 10.5	6.6 ± 9.2	0.2 ± 3.0	1.3 ± 2.7
***Pitch***	6.8 ± 4.9	8.1 ± 3.1	-0.4 ± 2.3	1.3 ± 1.9
***Yaw***	0.8 ± 7.6	5.8 ± 5.8	-0.3 ± 5.7	2.1 ± 5.3

**Fig 7:**
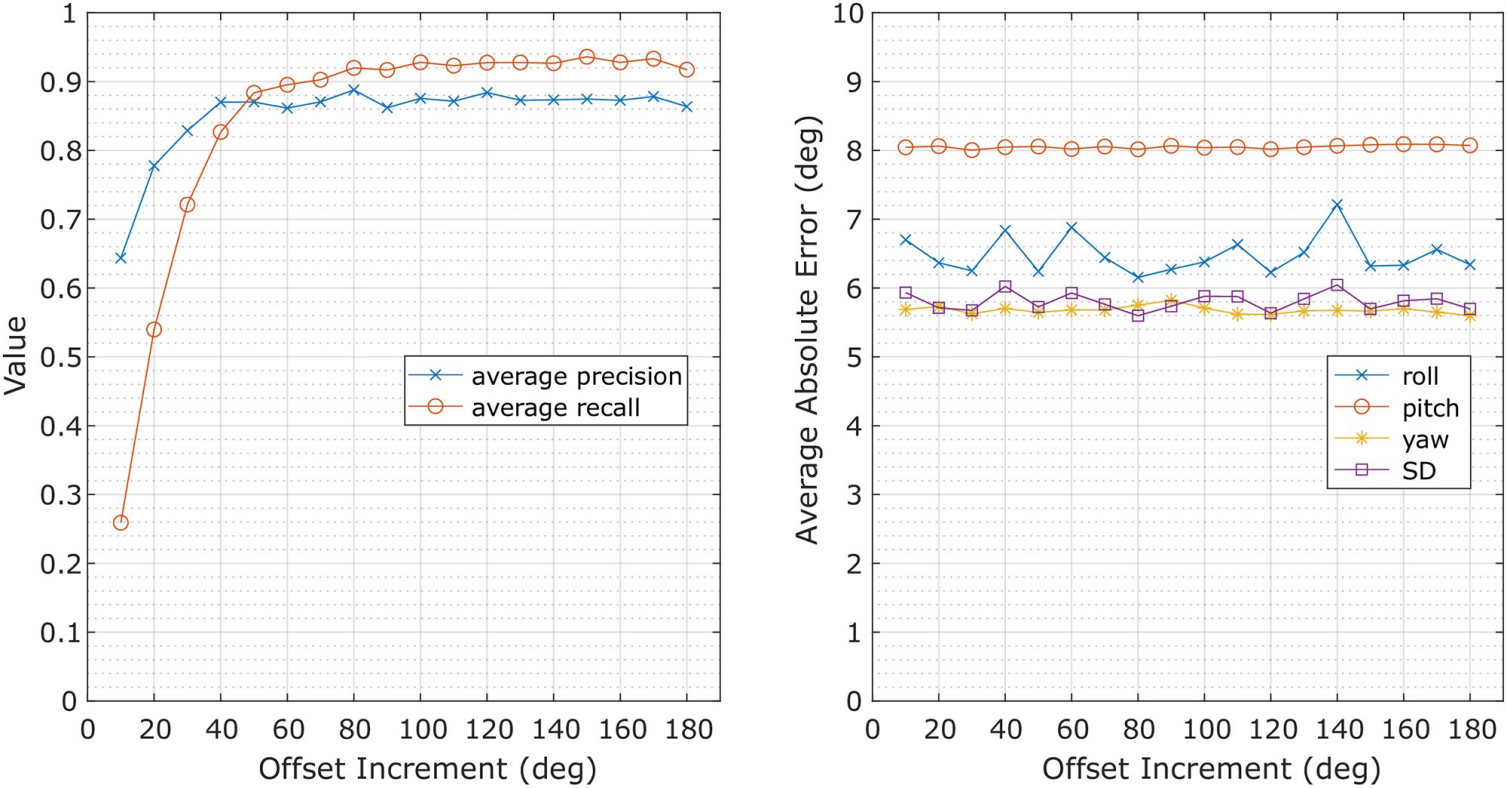
Average precision and recall of tag shift detection (left) and the average absolute error of the calculated animal pose after tag orientation correction (right) over simulated tag shifts with random direction and fixed degrees (*x*-axis). SD denotes the average of the standard deviation values for roll, pitch, and yaw at each offset increment.

To assess the sensitivity of the proposed shift method (**Objective** **4**), the computation experiments were repeated with virtually injected tag rotations that had random directions and **fixed** degrees. The average precision and recall for the detection of these injected tag shift instances are shown in Fig 7-Left. With a 10 degrees rotation offset, less than 30% of the injected shifts were detected, while with a 20 degrees offset, ∼54% of the shifts were detected with a detection precision of ∼79%. When the offset reached 30∼40 deg, the method’s performance became more reliable, with a recall of 70%∼80% and precision of ∼85%. Offsets of 50 degrees and above resulted in the performance converging to ∼90% precision and recall, respectively. Fig 7-Right presents the average absolute errors of the calculated poses after rotation correction for each of the data segments transformed by a rotation. The errors demonstrate similar values across all rotation offsets, with the average error for the roll of ∼6.5 degrees, the pitch of ∼8.0 degrees, and yaw of ∼5.7 degrees. The average standard deviation was ∼5.8 degrees.

**Fig 8:**
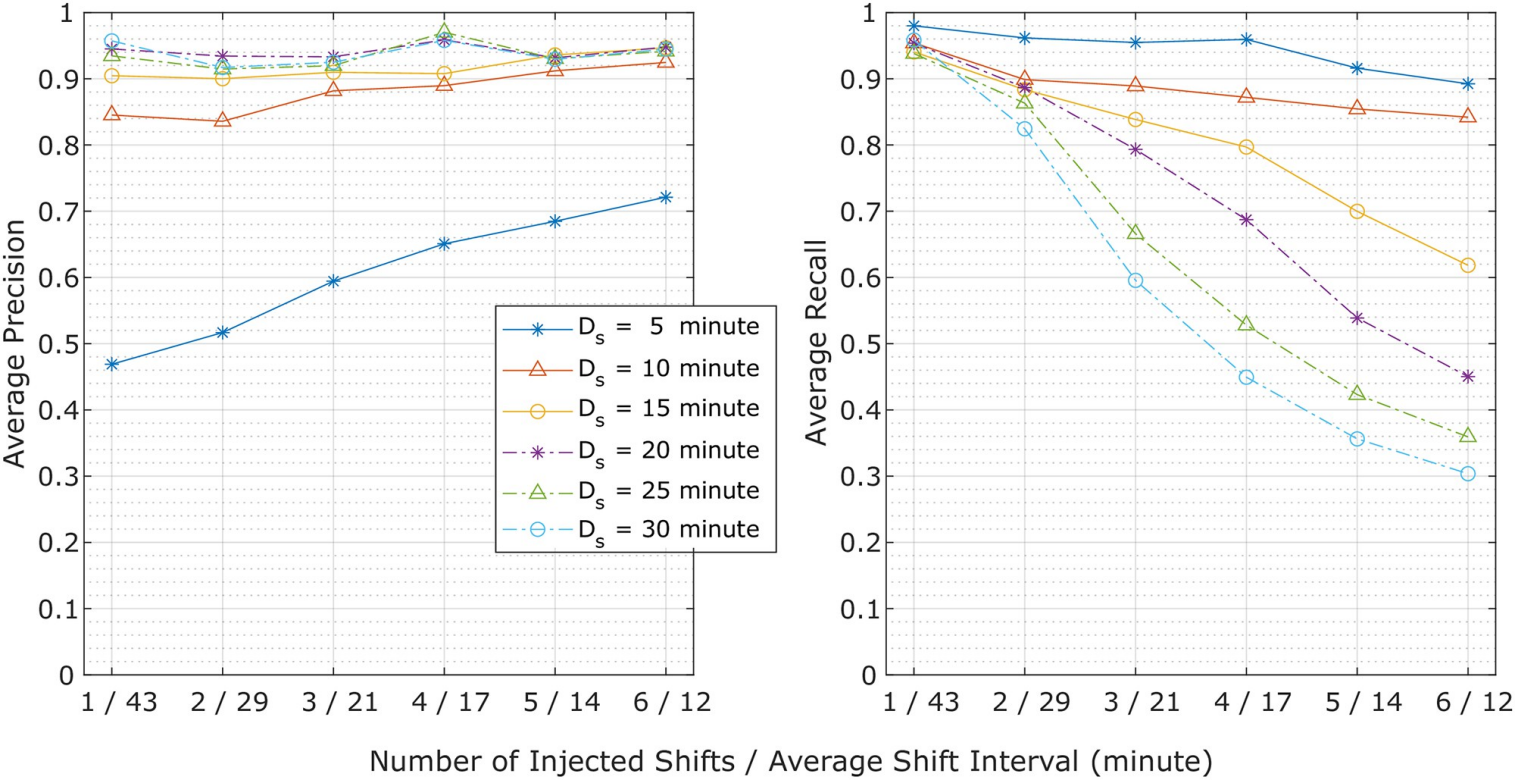
Average precision (left) and recall (right) of tag shift detection performance during simulation plotted against a varying number of injected shifts (or equivalently, the average shift interval). Shift detection performance at specific segment durations (*D*_*s*_) is demonstrated by the individual curves in the plot.

Fig 8 demonstrates the average precision and recall of tag shift detections using different segment duration settings *D*_*s*_ in response to a varying number of injected shifts *k* (or equivalently, the average shift interval, **Objective** **5**). The average precision across all *D*_*s*_ choices was between 0.83 and 0.97, except when *D*_*s*_ = 5 minutes, where the average precision increased from 0.47 to 0.72 as the number of injected shifts increased. The average recall of the different *D*_*s*_ values demonstrated a decreasing trend as the number of injected shifts increased, where the drop was more significant with larger *D*_*s*_ choices. However, the average recall values remained above ∼0.8 when the specified *D*_*s*_ was smaller than the average shift interval.

**Fig 9:**
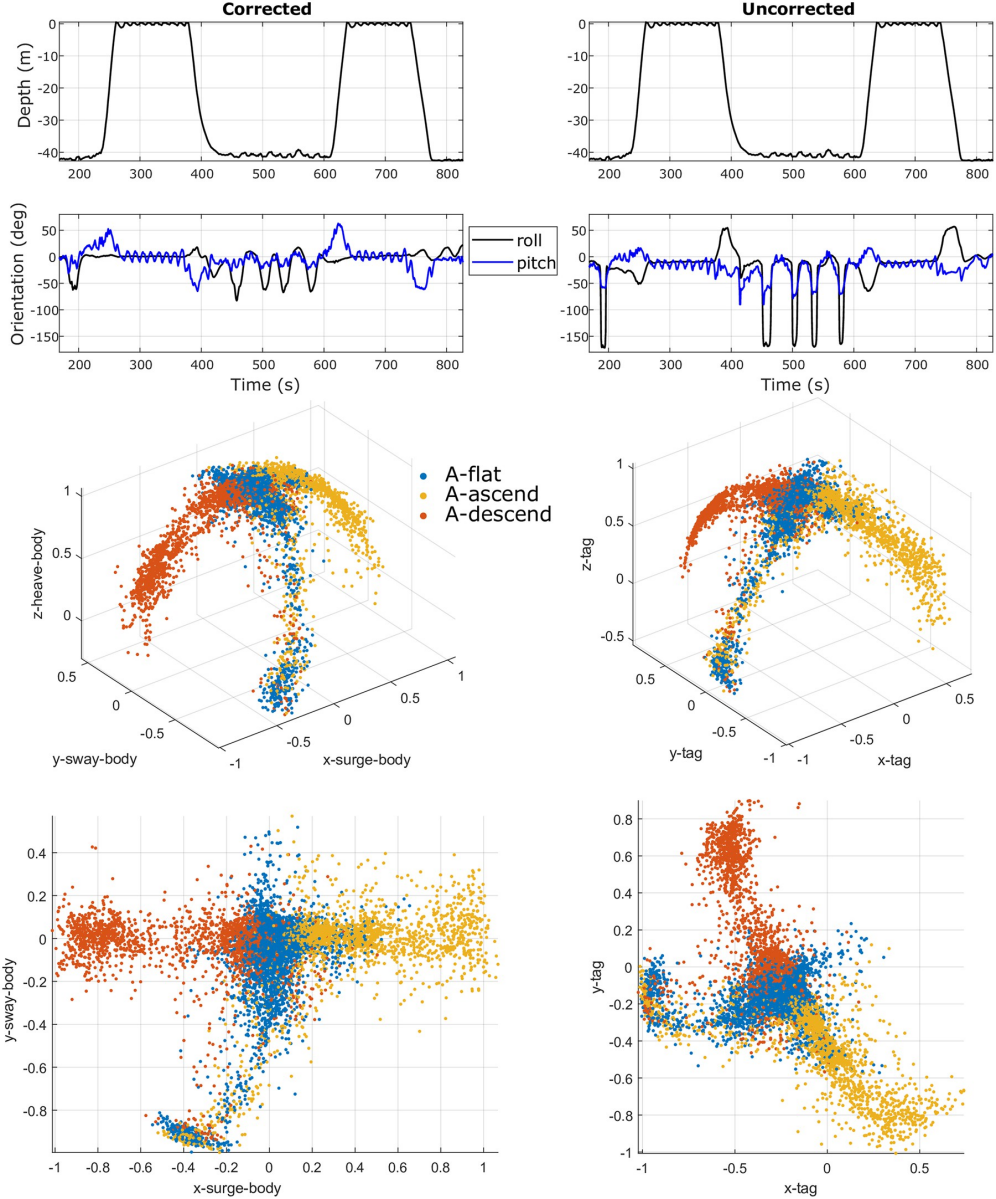
Representative data from a **humpback whale**, with the tag’s orientation corrected (left column) and uncorrected (right column). The first row shows the depth measurement from the pressure sensor; the second row presents the roll and pitch estimation made by the corrected (left) and uncorrected (right) tag data; the third and fourth rows illustrate the associated **orientation spheres** in a 3D view (third row) and a top-down view (fourth row).

**Fig 10:**
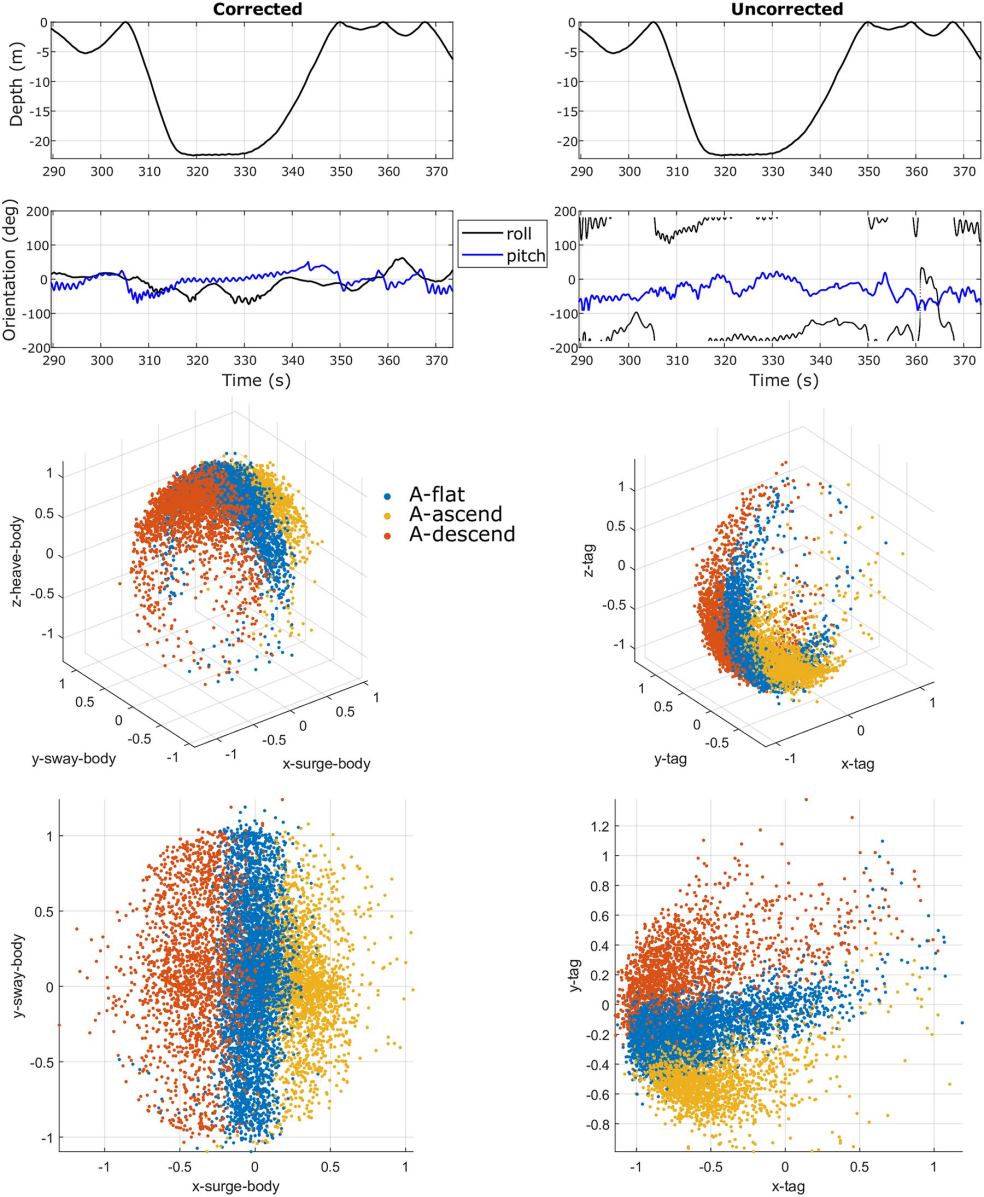
Representative data from a **beluga whale**, with the tag’s orientation corrected (left column) and uncorrected (right column). The first row shows the depth measurement from the pressure sensor; the second row presents the roll and pitch estimation made by the corrected (left) and uncorrected (right) tag data; the third and fourth rows illustrate the associated **orientation spheres** in a 3D view (third row) and a top-down view (fourth row).

The shift method’s performance on free-ranging animal data was examined qualitatively by examining the data after a shift correction was applied (**Objective** **6**). As an example, consider a few signal features from the corrected humpback whale data that can be identified in Fig 9-Left Column. First, corrected animal data showed a positive pitch during ascending and a negative pitch during descending. Second, corrected animal data showed a neutral roll angle (zero degrees) when the animal was at the surface. Third, the corrected pitch angle was zero-centered when at the surface. These are signal features commonly used in the literature [1] to inspect humpback whale data correctness. The orientation spheres (Fig 9-Bottom Two Rows), which visualize the animal’s orientation distribution (introduced with more detail in Fig 2), were examined visually for correctness. Plots from uncorrected data (Fig 9-Right Column) are shown for comparison. Similarly, Fig 10 presents the corrected and uncorrected example data from the beluga whale.

**Fig 11:**
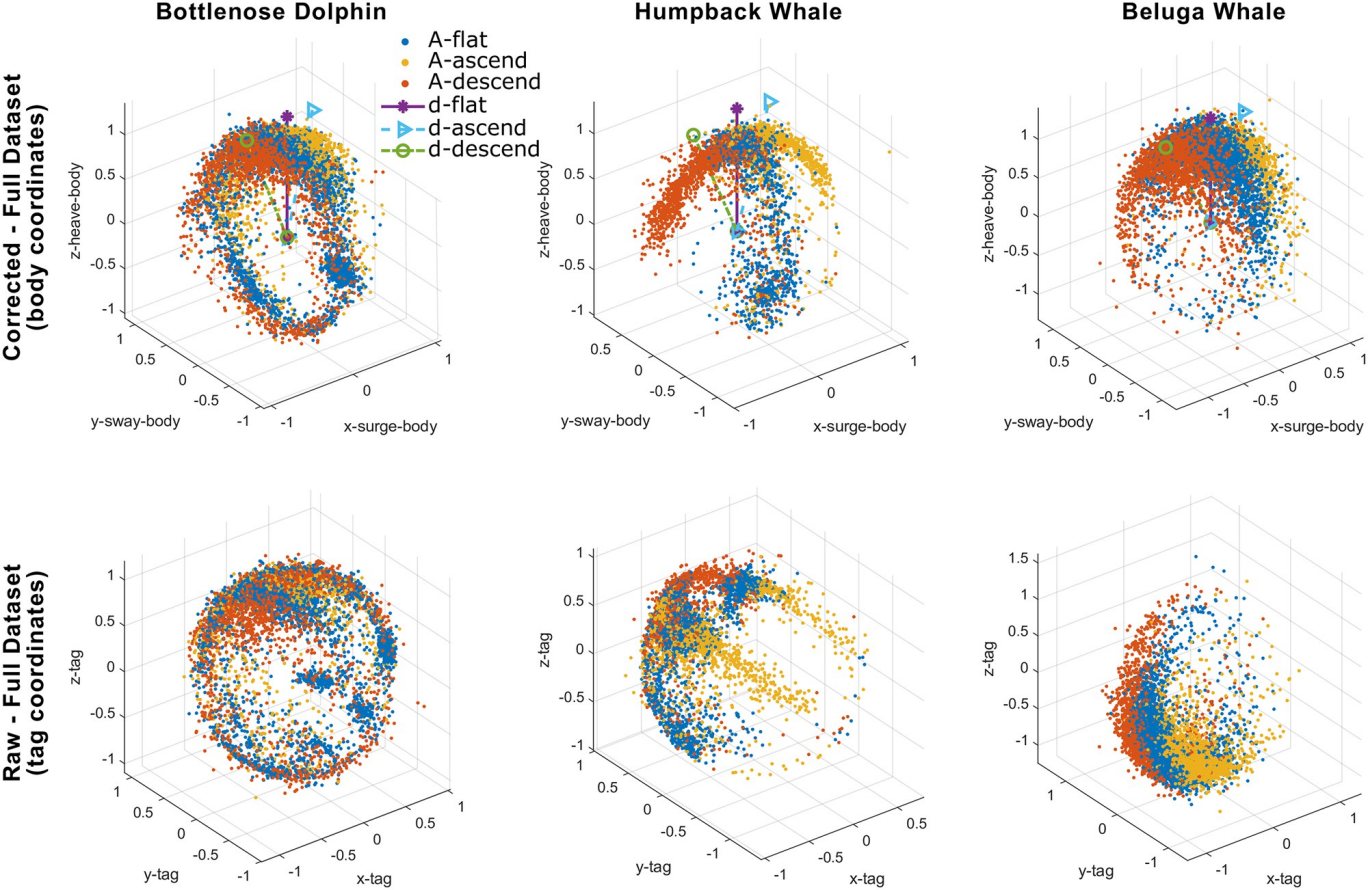
**Orientation spheres** for a bottlenose dolphin (left column), a humpback whale (center column), and a beluga whale (right column). The plot of accelerometer data clustered by depth speed is referred to as an **orientation sphere**. After detecting tag shifts and correcting tag orientation misalignment, the top row of subplots presents the orientation spheres in the animal’s body coordinates. The bottom row shows the data in the tag coordinates before any correction. The dolphin dataset (left column) contains four simulated tag shifts. The humpback whale dataset (center column) has five detected tag shifts. The beluga dataset (right column) is not aligned with the animal but contains no detected tag shifts (i.e., no relative motion between tag and animal was detected).

Fig 11 presents the orientation spheres for an example bottlenose dolphin dataset (left column), a humpback whale dataset (center column), and a beluga whale dataset (right column, **Objective** **6**). The top row shows the orientation spheres after applying the shift corrections. The bottom row provides the raw data before tag shift detection and orientation corrections. The raw dataset of the bottlenose dolphin (bottom left) contains four simulated tag shifts, while six tag shifts were detected from the humpback whale dataset (bottom center). The raw tag data from the beluga whale (bottom right) indicates that the tag was not aligned with the animal but contains no detected tag shift (i.e., no relative motion between tag and animal was detected).

**Fig 12:**
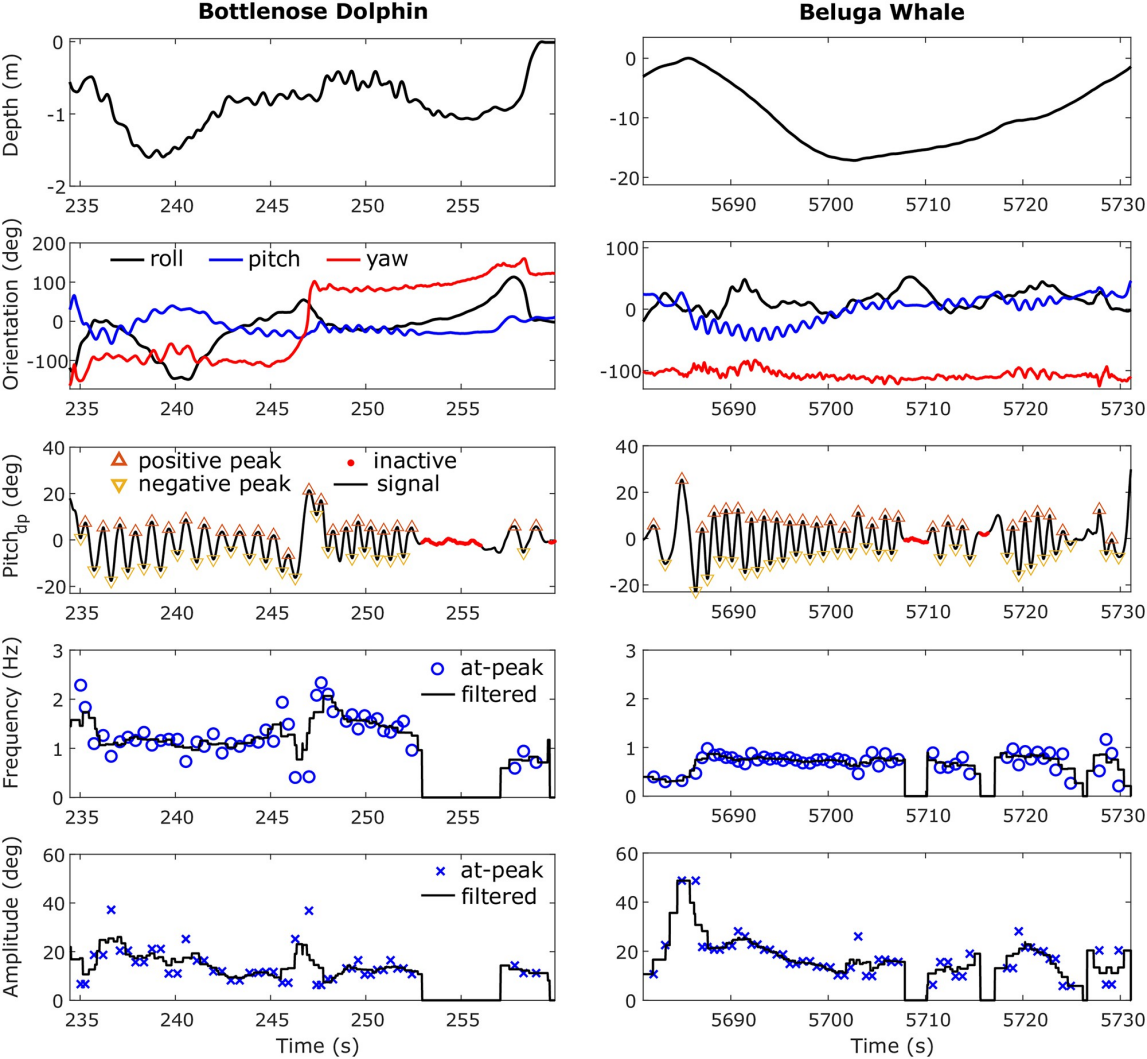
Example sections of data from a bottlenose dolphin (**left**) and a beluga whale (**right**) with the animal’s gait parameterized via **dynamic pitch** (*pitch*_*dp*_). When the animal has a big roll angle (e.g., during the time around 240 seconds in the bottlenose dolphin dataset), the fluking ‘signature’ (i.e., the sinusoidal fluctuations in each signal channel) transfers from pitch to yaw in the pose estimations. *Pitch*_*dp*_ is used to have a pose invariant descriptor of the gait of the animal, that is, with respect to the animal rather than the environment. Inactive swimming periods (e.g., gliding) are automatically identified in *pitch*_*dp*_ while fluking frequency and amplitude are calculated from *pitch*_*dp*_, which can be used for further gait analysis.

Fig 12 presents example sections of data from one of the bottlenose dolphin (left) and the beluga whale (right) datasets, where the pose of the animal was calculated from the corrected body coordinates’ data, and the gait of the animal was characterized using the *pitch*_*dp*_ (**dynamic pitch**) of the animal. The mean fluking period of the bottlenose dolphin was 1.0 s with an average amplitude of 14.1 degrees. The mean fluking period of the beluga whale was 1.6 s, with an average amplitude of 16.8 degrees. For the humpback whale dataset, the mean fluking period was 6.7 s with an amplitude of 12.2 degrees.

## Discussion

The automatic shift detection algorithm is the first of its kind and is an important step in the development of algorithms that will streamline the analysis of biologging data. Simulated datasets were derived from bottlenose dolphins tag data to provide an extensive validation dataset that covered a wide variety of tag shifts. An additional benefit of the simulated datasets is that ground truth measurements were immediately available for analysis. This method demonstrated a detection precision of 0.87 and a recall of 0.89 with an average precision error of 37.5 seconds with the simulated data. When the algorithms were applied to the original data that contained no tag shift, a false detection only occurred every 8.7 hours on average. One reason for the missed detections in the simulated data was the close temporal proximity of the simulated tag shifts.

While the method may not detect multiple shifts within a segment, in practice tags tend to shift *occasionally* during a deployment. During parameter selection, duration *D*_*s*_ was chosen to reduce false positives yet maintain recall. We heuristically determined the duration of a signal segment (*D*_*s*_) to be 10 minutes for bottlenose dolphins and 15 minutes for beluga whales and humpback whales. As demonstrated in Fig 8, if the duration of a data segment is too short, there is not enough data to form a meaningful orientation sphere for shift detection. While if the duration of a data segment is too long, the method may not detect temporally close shifts. In practice, a larger value (e.g., 30 minutes) may be selected and incrementally reduced if two or more shifts are detected within two segments.

Missed detections also occurred when the tag shift was too small to be detected. Sensitivity to the magnitude of the shift is presented in Fig 7 and indicates that the method may not perform well for changes in orientation of fewer than 40 degrees. One way to improve performance would be to use a lower detection threshold for inlier percentages, but this would introduce more false positives. However, these false positives may be preferred to missed detections for this application. The recall could also be improved by taking events associated with high acceleration impact on the tag, such as an impact from a conspecific animal in the group, into account [1].

False positives were generated when the animals switched from one gait to another (very different) gait. For example, the detector could be falsely triggered when the animal changed from a gait without any roll to a swimming gait with rolling. Significant gait changes effectively violate our third assumption: continuity of gait patterns between segments. This situation occurred once per 8.7 hours in the validation datasets and did not pose a major problem for the overall correction method. Individually correcting two segments separated by a false positive is less optimal than correcting them jointly as one segment, given that the amount of data for each correction decision is reduced. But this would not necessarily reduce the final data correction performance since the two data segments would still be processed and corrected.

To decouple the evaluation of tag shift detection and tag orientation correction, the tag orientation correction method was applied to each data segment to determine the rotational transformation. Animal pose (roll, pitch, and yaw) was calculated after orientation correction and then compared with the poses computed using the original (un-rotated) data. The average of the differences between the two were within 11 degrees in all cases (Table 1A vs. 1B and Fig 7-Right). This result indicates that the tag orientation correction method is robust to tag location as long as the data segment does not contain a tag shift. One thing to note is that the error associated with pitch are higher than roll and yaw by a few degrees (Table 1A vs. 1B and Fig 7-Right). This difference is likely due to an initial bias in the data. The pose calculated from the original ‘aligned’ data resulted in pitching angles centered around negative 6∼7 degrees instead of 0 degrees. Tags were ‘aligned’ with the animal’s body when placed between the dorsal fin and blowhole (Fig 1-Left). But tapering of the animal’s body may have resulted in a small initial negative pitch of the tag, creating a negative bias in the ‘ground truth’ data. As such, the pitching angle estimated by the tag orientation correction could be a better estimate than what was originally calculated from the ‘aligned’ tag data.

The animal poses calculated using the proposed method (accelerometer and magnetometer only) in this work were compared with an established filtering approach using the full set of IMU measurements (accelerometer, magnetometer, and gyroscope [24], Table 1B vs. 1C). The resulting average differences between these two methods were within 3 degrees. The error in the pose calculated using the proposed estimation method is likely due to errors in the estimate of gravitational acceleration. In this work, low-pass filtered 3-axis accelerometer data were used to estimate the gravitational acceleration (***A***^(*tag*)^) for the pose calculation. However, this is an inaccurate estimation in practice since the accelerometers measure both gravity and the animal’s specific acceleration (i.e., the animal’s physical acceleration). This motion affects accelerometer measurements in 3 ways:

(a1) Measurements associated with an animal’s specific acceleration. Since normally, the animal would not maintain a constant acceleration for more than a few seconds, all specific accelerations are considered high frequency.
(a2) Measurements associated with gravity, driven by **high** frequency body orientation changes.
(a3) Measurements associated with gravity, driven by **low** frequency body orientation changes.

Because measurements associated with gravity (a2 & a3) are needed for estimating animal pose, a1 needs to be decoupled from a2 and a3. An accurate decoupling is not possible using an accelerometer alone. But because these specific accelerations created by animal motion are generally much smaller than gravity, a low-pass filter can be applied to attenuate a1 and a2 to approximately filter out specific acceleration measurement (a1) from gravity (a2 & a3).

To better decouple measurements of specific acceleration (a1) from gravity (a2 & a3), López et al. [34, 35] presented an approach that uses magnetometer data to directly estimate the high-frequency ‘pitching’ motion of the animal. This approach uses magnetometer data to assist in finding a2, with a3 obtained via low-pass filtering. Then, a1 is calculated by subtracting a2 and a3 from the accelerometer measurement. The method works well in decoupling specific acceleration and orientation for most cases. However, because the method assumes high-frequency changes in body orientation occur only in the pitching plane, the calculations can be unstable when the animal turns or rolls. We recommend using the method presented by López et al. for animals with low turning or rolling rates and have included the corresponding code in the toolbox proposed in this work. For tags with gyroscopes, accelerometers, and magnetometers, we recommend using a gradient descent-based filtering approach by Madgwick et al. [24] to estimate the tag’s orientation wrt the world. The method uses the gyroscope to directly estimate a2, thus reducing uncertainty in the final orientation estimate. A wrapper for the Madgwick’s approach is also included in the proposed toolbox. The gait characterization method presented in this work complements the above approaches and provides both frequency and amplitude estimations that are stable and invariant to the pose of the animal.

While the proposed approach corrects the relative orientation of the tag with respect to the animal, the actual location of the tag (e.g., back vs. peduncle) could still affect tag measurements and estimates of gait parameters like fluking amplitude. For example, a tag located closer to the fluke will have a higher estimated fluking amplitude than a tag located near the dorsal fin for the same gait. So, estimates of fluking amplitude may be a good parameter to characterize the behavior of an individual animal (e.g., comparing fluking amplitude between descent and ascent) but not for global comparisons (e.g., comparing one animal to another, with different tag locations). In contrast, fluking frequency and period are not affected by the tag’s location.

The orientation spheres (e.g., Figs 2 and 11) can also serve as a visualization tool for animal behavior studies. For example, a clear sign of lateralized behavior [36] appears in the orientation sphere of the humpback whale (Fig 9-Left Column and Fig 11-Center Column), with an unbalanced right ‘wing’ corresponding to a left roll (i.e., negative roll) during foraging at the ocean bottom. While the humpback whale did not roll much during ascent/descent in this example, the bottlenose dolphin (Fig 11-Left Column) and beluga whale (Fig 10-Left Column and Fig 11-Right Column) rolled more frequently during similar portions of a dive. Even though the beluga rolled frequently, it hardly ever rolled completely upside down, which can be observed from the empty bottom of the orientation sphere. Unlike the beluga, the dolphin data shown in Fig 11 indicates that the animal rolled all the way around during the studied period. In this regard, the presented orientation sphere, to a degree, resembles the *m-sphere* generated using magnetometer data in the work by Williams et al. [37] as well as the *o-sphere* for visualizing an animal’s head orientation in the work by Wilson et al. [31].

It is important to note that the method leverages patterns in swimming movement data, and does not directly apply to situations when the animal is still (e.g., resting) for long periods of time. Additional software modules should be implemented to handle such special situations. Resting could be automatically detected using the accelerometer measurements, and tag orientation correction that was identified before the resting could be applied to the resting periods. Another aspect for future investigation is the detection and management of discrete consecutive shifts that can not be treated as *one* time instance, and deployments where the tag is constantly shifting. Nevertheless, if a sequence of consecutive shifts happened within a minute or two in a multiple-hour-long dataset, the sequence of shifts could be treated as one shift, per the resolution requirement of the application.

The presented method demonstrated good performance across the validation datasets with manually tuned lower-level parameters. These parameters could be systematically investigated further in future work. Future work could also include a formal comparison between the pose estimates generated using the proposed approach and conventional methods [1]. Results and insights from these assessments could then be used to further develop and improve the automated method. In practice, the method could be used alongside the conventional methods to help the human expert find the dives when a tag shift might happen and suggest shift times that could be confirmed by the user.

## Conclusion

This paper presents an automated data processing framework (and software) that takes advantage of the common characteristics of cetacean pose and gait during swimming to estimate the pose of the animal and analyze gait from biologging tag data. The proposed approach: (1) Automatically identifies tag shifts (a change in tag orientation with respect to the animal) during a deployment; (2) Calculates the relative orientation of the tag wrt the animal’s body for a given data segment during the deployment; (3) Provides metrics for gait analysis that are stable and invariant to pose and tag orientation. Biologging tag data from bottlenose dolphins, a humpback whale, and a beluga whale were used to validate and demonstrate the proposed approach. Results show that the average relative orientation error of the tag wrt the dolphin’s body after processing was within 11 degrees in roll, pitch and yaw directions. In addition, the average precision and recall when identifying a tag shift were 0.87 and 0.89, respectively. Examples of the resulting pose and gait analysis demonstrate the potential of this approach to enhance movement analysis and animal behavioral studies. The proposed analysis approach and software will facilitate the use of biologging tags to study cetacean locomotion and behavior. The method and software are applicable to cetacean data from any tag platform that uses an accelerometer, magnetometer, and pressure sensor.
